# Neutrophil Extracellular Traps (NETs) in Severe SARS-CoV-2 Lung Disease

**DOI:** 10.3390/ijms22168854

**Published:** 2021-08-17

**Authors:** Monika Szturmowicz, Urszula Demkow

**Affiliations:** 1 I Department of Lung Diseases, National Tuberculosis and Lung Diseases Research Institute, Plocka 26, 01-138 Warsaw, Poland; monika.szturmowicz@gmail.com; 2 Department of Laboratory Diagnostics and Clinical Immunology of Developmental Age, Medical University of Warsaw, Żwirki i Wigury 63A, 02-091 Warsaw, Poland

**Keywords:** NETs, SARS-CoV-2, COVID-19 disease, acute lung injury, immunothrombosis, cytokine storm

## Abstract

Neutrophil extracellular traps (NETs), built from mitochondrial or nuclear DNA, proteinases, and histones, entrap and eliminate pathogens in the course of bacterial or viral infections. Neutrophils’ activation and the formation of NETs have been described as major risk factors for acute lung injury, multi-organ damage, and mortality in COVID-19 disease. NETs-related lung injury involves both epithelial and endothelial cells, as well as the alveolar-capillary barrier. The markers for NETs formation, such as circulating DNA, neutrophil elastase (NE) activity, or myeloperoxidase-DNA complexes, were found in lung specimens of COVID-19 victims, as well as in sera and tracheal aspirates obtained from COVID-19 patients. DNA threads form large conglomerates causing local obstruction of the small bronchi and together with NE are responsible for overproduction of mucin by epithelial cells. Various components of NETs are involved in the pathogenesis of cytokine storm in SARS-CoV-2 pulmonary disease. NETs are responsible for the interplay between inflammation and thrombosis in the affected lungs. The immunothrombosis, stimulated by NETs, has a poor prognostic significance. Better understanding of the role of NETs in the course of COVID-19 can help to develop novel approaches to the therapeutic interventions in this condition.

## 1. NETs—Introduction

Neutrophils are the first-line defense cells eliminating microorganisms through unspecific mechanisms, such as oxidative stress, and the production of various antimicrobial factors [1]. The main strategies used by neutrophils to control infections include phagocytosis or release of granule-derived cytotoxic mediators [2]. The formation of neutrophil extracellular traps (NETs) has recently been proposed as a distinct mechanism of innate immune response, by which neutrophils localize and kill pathogens. 

## 2. NETs—Structure, Mechanisms of Formation, and Pathogenic Effects

NETs are large, extracellular, web-like structures composed of DNA fibers decorated with various antimicrobial factors such as: citrullinated histones, defensins, neutrophil elastase (NE), and myeloperoxidase (MPO) [3,4]. NETs are very efficient in capturing and destroying pathogens, thus localizing infection and limiting the spread of bacteria, viruses, or fungi [4]. 

**Figure 1:**
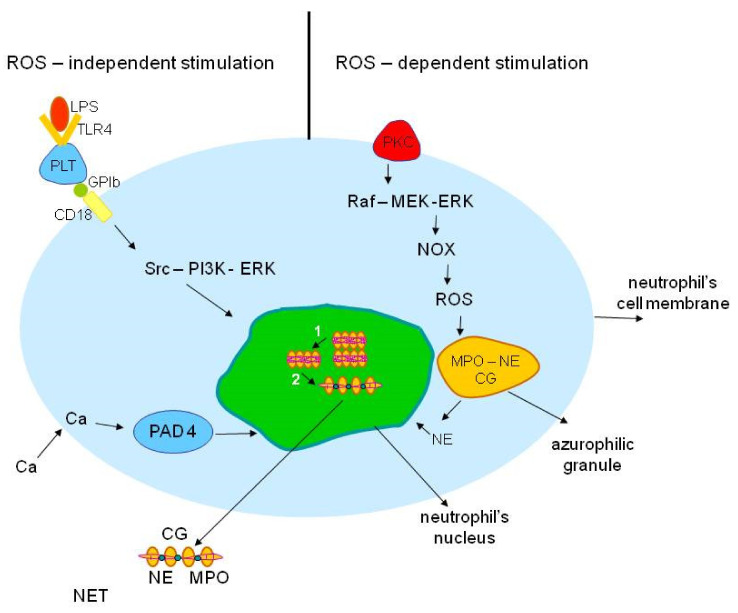
Mechanisms of NETs formation. Classically, NETs production is initiated via reactive oxygen species (ROS)-dependent activation of neutrophils. The inducting substance binds to protein C kinase receptor (PKC) localized on the neutrophil’s cell membrane. Activation of PKC initiates MAPK pathway which results in NADPH-oxidase (NOX) activation and ROS production. ROS influx to azurophilic granules causes the detachment of neutrophil elastase (NE) from myeloperoxidase (MPO), with subsequent NE and MPO release and migration to the neutrophil’s nucleus. NE triggers histone degradation and chromatin decondensation, further enhanced by MPO. Peptidylarginine deiminase 4 (PAD 4)-mediated histone citrullination subsequently leads to chromatin decondensation. Finally, nucleus swelling and the release of NETs (complexes of nucleic fibers and citrullinated histones, coated, among others, with NE, MPO, and CG, are observed. Generation of ROS-independent NETs via Toll-like receptor 4 (TLR4) has also been demonstrated in calcium-mediated and platelet-stimulated NETosis. Legend: Ca—calcium, CG—cathepsin, CD18—ß_2_-integrin, GPIb—glycoprotein Ib, LPS—lipopolysaccharide, MPO—myeloperoxidase, NE—neutrophil elastase, NET—neutrophil extracellular trap, NOX—NADPH oxidase, PAD 4—peptidylarginine deiminase type 4, PKC—protein kinase C, PLT—platelet, Raf-MEK-ERK—MAP kinase pathway, ROS—reactive oxygen species, TLR4—Toll-like receptor 4, 1—chromatin decondensation, 2—histone citrullination.

Classically, NETs production is initiated via reactive oxygen species (ROS)-dependent activation of neutrophils (Figure 1). NADPH oxidase (NOX) generates ROS production, which in turn induces migration of myeloperoxidase (MPO) and neutrophil elastase (NE) from azurophilic granules to the nucleus, where NE triggers histone degradation and chromatin decondensation, further enhanced by MPO [5]. Citrullination and chromatin decondensation are mediated by the enzyme peptidylarginine deiminase 4 (PAD4). PAD4-mediated modification of nuclear histones by converting arginine residues to citrullines, together with activation of the Raf-MEK-ERK signal transduction pathway, subsequently leads to chromatin decondensation [6]. The final steps of the process include nuclear swelling, destruction of the nuclear envelope, extrusion of the decondensed chromatin, and cell membrane rupture.

Classically, NETs release results in cell death [7]. Nevertheless, a non-suicidal pathway of NET formation was also described, where the cell remains intact and normal cellular functions of neutrophils, such as chemotaxis and phagocytosis, are still carried out [7]. Recent studies suggest an increasingly more complex picture, in which the requirements for NADPH-oxidase, ROS, MPO, NE, and PAD enzymes are influenced by the specific triggers of NETs [6,8]. ROS generation has been shown to be sufficient for NET formation only when following autophagy signaling pathways [8]. The requirement for both autophagy and NADPH-oxidase activity in the course of NETosis has recently been confirmed in *Candida-albicans*-induced NETs, as well as in sepsis-triggered NETosis [9]. Generation of ROS-independent NETs via Toll-like receptor 4 (TLR4) has also been demonstrated in platelet-stimulated NETosis in the course of sepsis [10]. 

### 2.1. NETs in the Course of Severe Infections

A number of highly significant associations between NETs release and both bacterial and viral infections have been found. NETs play a major role in preventing dissemination of pathogens. NETs are able to entrap and eliminate pathogens. As an example, this structure is believed to sequester and inactivate viruses as confirmed for influenza [11,12]. In the course of an infection, NETs production is triggered not only by microorganisms, but also by various pro-inflammatory mediators: tumor necrosis factor alpha (TNF-α), interleukin-8 (IL-8), nitric oxide, activated platelets, activated endothelial cells, and a large panel of auto-antibodies. The beneficial role of NETs in the innate immune response to invading microorganisms coexists with its detrimental effects: the promotion of tissue damage, thrombosis, and autoimmunity [4]. NETs components, in particular histones, DNA fibers, and antimicrobial proteins, significantly contribute to lethality in generalized infections [13]. 

### 2.2. NETs and Thrombosis

The interplay of inflammation and thrombosis is well established. NETs formation is a key factor contributing to the occurrence of thrombotic events in the course of inflammatory processes. Several mechanisms of NETs-induced thrombosis were proposed [13]. 

NETs promote adhesion of activated platelets and red blood cells to nucleic acid filaments. DNA fibers serve as a milieu concentrating coagulation factors and activators participating in the course of intravascular thrombus initiation and propagation [14]. 

Negatively charged DNA strands and NE are potent pro-coagulants. NETs have been shown to activate coagulation factors (for example, contact factor XII and factor X), thus participating in disseminated intravascular coagulation (DIC) and fibrin deposition [15,16]. Moreover NETs have been shown to co-localize with von Willebrand factor, which constitutes a bridge between NETs and the endothelium [17], as well as with tissue factor (TF), thus initiating the extrinsic coagulation pathway [18]. 

Skendros et al. found that the presence of NETs was positively correlated with thrombin-antithrombin activity [19]. Ducroux et al. have shown that NETs components have anti-fibrinolytic effect, so recombinant tissue plasminogen activator (rtPA) therapy alone may not be sufficient to dissolve clots entangled in NETs [20]. Additionally, the pro-thrombotic milieu is influenced by the increase of plasminogen activator inhibitor-1 (PAI-1), and inhibition of anticoagulant protein S [21]. 

Furthermore, it has been observed that NETs components, including histones, trigger both direct and complement-induced platelets activation [22,23,24]. It was demonstrated that histone infusion resulted in platelets aggregation and the formation of microthrombi in the mice model of sepsis [15]. Moreover, it was found that infused histones are toxic for endothelial and epithelial cells and this effect is mediated via TLR2 and TLR4 [25]. Elaskalani et al. showed that aggregation of platelets could also be induced by cathepsin G (CAT G), which is the other catalytic component of NETs [26]. NETs-induced platelets aggregation may explain the thrombocytopenia observed in patients with severe infections. 

Ligation of TLR4 by lipopolysaccharides in narrow vessels induces platelet binding to accumulating neutrophils, thus promoting NETosis locally. ß-defensin 1 (bactericidal component of platelets) can induce NETs production. Platelet-neutrophil interactions leading to NETs formation may play an important role in endothelial cell and tissue damage, not only in the course of sepsis, but also in other chronic inflammatory disorders [4]. 

Mutual interactions of the coagulation system and NETs, resulting in pro-thrombotic activity, are referred to as immunothrombosis [27].

## 3. SARS-CoV-2 Lung Disease

### 3.1. Pathogen Characteristics and Mechanisms of Infection

Severe acute respiratory syndrome coronavirus 2 (SARS-CoV-2) is a new type of coronavirus responsible for the worldwide pandemic announced by the WHO in March 2020 [28]. Since the description of the first cases of severe COVID-19 pneumonia in December 2019 in Wuhan, China, the number of patients with confirmed infection has grown up to 107 838 255 with 2 373 398 deaths worldwide (as of 13 February 2021) [29]. 

In the majority of patients infected with SARS-CoV-2, the disease course is benign, presenting as self-limiting inflammatory syndrome of the upper respiratory tract [28,30]. Nevertheless, severe pneumonia develops in 15–20% of patients, and acute respiratory distress syndrome (ARDS) is diagnosed in about 5% of them [31]. In 1–2% of COVID-19 patients, the course of the disease is fatal [32]. The most frequent causes of death in the acute phase of COVID-19 include ARDS and multi-organ failure, as well as venous and arterial thrombosis [30].

SARS-CoV-2 is a single-stranded large RNA virus, with a genome of 27–32 kB [33]. It is composed of four main structural genes encoding the nucleocapsid protein (N), the spike glycoprotein (S), the small membrane protein (SM), and the membrane glycoprotein (M). SARS-CoV-2 infects host cells via angiotensin-converting enzyme 2 receptor (ACE 2). ACE 2 receptor is a metallopeptidase present on the cell surface of bronchial epithelial cells, pneumocytes type 1 and 2, alveolar macrophages, and pulmonary endothelial cells [34]. The subunit S1 of viral protein S binds to ACE 2 receptors on respiratory tract epithelial cells and alveolar pneumocytes, and after the dissociation of S1 from S2 by the serine protease, the fusion of the virus with the host cell occurs [21,30]. 

During the preliminary phase of SARS-CoV-2 infection, immunological tolerance develops, manifesting with inhibition of interferons by SARS-CoV-2 proteins and enabling viral replication and infection of a growing number of respiratory cells [21,35].

### 3.2. Lung Histopathology in COVID-19 Disease

The post-mortem studies of lung tissue samples documented the nature of the pathologic process responsible for lethal pulmonary COVID-19 disease. The predominating pattern of lung injury was diffuse alveolar damage (DAD) [36,37]. The exudative form of DAD was described as pneumocytes’ necrosis, exudation of blood proteins to lung alveoli with subsequent hyaline membranes formation, and interstitial and intra-alveolar lung edema [36,37]. The pathologic analysis of lungs of COVID-19 victims revealed a heterogenic process, with the areas of exudative DAD and other lung fields affected with the proliferative phase of DAD characterized by pneumocyte type-2 hyperplasia and lung fibrosis [36,37]. An attempt to correlate the postmortem radiologic lung patterns and the results of histopathological examination was made. The presence of ground glass opacities correlated with the areas of exudative DAD, consolidating ground glass opacities—with proliferative DAD and radiologic honeycombing—with histopathological signs of lung fibrosis [37]. Lung fibrosis was confirmed on post-mortem examination in those patients who died of COVID-19 lung disease of at least 4 weeks duration. 

An important part of the pathogenic mechanisms in the course of SARS-CoV-2 infection of the lung is the formation of platelet-fibrin complexes in the small pulmonary arteries and thrombosis of pulmonary capillaries [36,38,39]. Mauad et al. found thrombosis of small pulmonary arteries in 90% of autopsies performed in COVID-19 victims [40]. This process was not dependent on COVID-19 disease duration. Moreover, the DAD with coexisting extensive thrombosis of small pulmonary arteries and capillaries was more characteristic for SARS-CoV-2-induced ARDS, compared to ARDS induced by other infective agents, such as influenza virus [37,41]. 

## 4. NETs in COVID-19 Lungs

Acute lung injury in the course of Sars-CoV-2 infection, and its most severe form—ARDS, is closely dependent on NETs formation within the lung tissue and lung vasculature. In addition, neutrophil activation and the formation of NETs have been described as major risk factors for mortality in COVID-19 patients [42]. The autopsy reports of patients who died from COVID-19 showed intensive neutrophil infiltration. Xu et al. confirmed a high expression of extracellular DNA components of neutrophil extracellular traps (NETs) in tissue samples from COVID-19 patients [43]. NETs have been found in the microvasculature of lung, kidney, and heart of COVID-19 victims, and were considered to contribute to immunothrombosis-mediated damage of these organs [44].

### 4.1. Pathogenesis of NETs in SARS-CoV-2 Infection

**Figure 2:**
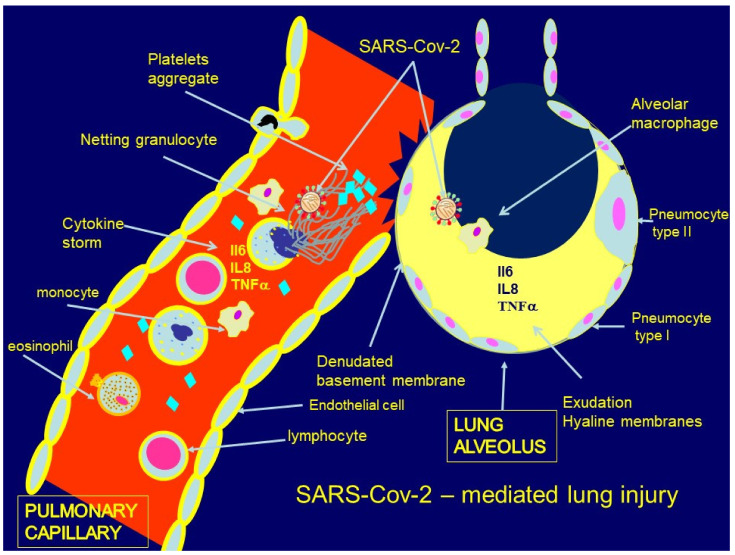
Figure 2 presents the pathogenesis of SARS-COV-2-mediated lung injury. The profound lung damage in the course of SARS-COV-2 is initiated by viral pneumonia. The process starts from the activation of the inflammatory response directed against the virus both in the pulmonary capillaries and within alveolar space. Inflammatory mediators disrupt the blood/alveolar barrier function. Excessive inflammatory response involving NETs formation, together with cytokine storm and in situ thrombosis in the pulmonary circulation, contributes to endothelial and alveolar damage. Specifically, greater permeability of the blood/alveolar barrier results in the accumulation of protein-rich alveolar exudation and hyaline membrane formation within alveoli. Moreover, increased endothelial permeability is responsible for leakage of fluid rich in inflammatory mediators and migration of immune cells, together with their products as NETs, platelets, and into the alveolar space. Once exudative fluid accumulates in the interstitium and alveoli, it causes impaired gas exchange, resulting in hypoxemia and ultimately acute respiratory failure.

Pathogenesis of lung injury associated with NETs in SARS-CoV-2 infection is presented in Figure 2. SARS-CoV-2 infects pulmonary cells expressing ACE 2 receptor, among them pneumocytes type 1 and 2, as well as pulmonary epithelial and endothelial cells [30]. The direct infection of neutrophils is also possible [30]. Viral S-protein cleavage, which is necessary to complete the fusion process, depends on the activity of serine protease in host cells. 

Induction of nuclear-factor-kB signaling cascade results in the excessive release of the inflammatory cytokines such as IL-1b, IL-6, IL-8, TNFα, and G-CSF. These mediators enable the progressive recruitment of various inflammatory cells from circulation and their influx into the lungs. Further recruitment of neutrophils is also possible due to G-CSF-induced maturation of bone-marrow-derived neutrophil precursors [35]. Both SARS-CoV-2 molecules and inflammatory cytokines, such as TNFα and IL-8, are capable of inducing neutrophil activation. 

Within the activated neutrophils, the production of NADPH-mediated ROS activates the endoplasmic core granules containing enzymes, among others, PAD-4. PAD-4 citrullination of DNA histones results in chromatin decondensation and release of NETs as complexes of chromatin, MPO, NE, and CAT G catalytic enzymes. ROS-independent pathway of NETs formation is also probably activated in the course of SARS-CoV-2 infection. 

Thus, the process of NETs formation in response to SARS-CoV-2 infection is controlled, among others, by the intensity of viral replication as well as the number of cells expressing ACE 2 receptor, as well as the activity of host cell serine protease and PAD-4 [30].

### 4.2. Mechanism of NETs-Related Lung Injury in SARS-CoV-2 Disease

NETs have been shown to participate in lung injury through various mechanisms. Endothelial and lung epithelial cell death, intravascular thrombus formation, and lung injury have been reported to be caused by NETs and their components [45]. Protein components of NETs, particularly histones, induce epithelial and endothelial cell death in a mouse model of LPS-induced acute lung injury [45]. 

The DNA threads localized in small bronchi form large complexes with mucin, causing local obstruction. Blocked airways constitute a suitable environment for bacterial growth and colonization [46]. 

Both NET-bound proteases, i.e., MPO and NE, degrade the glycosaminoglycan heparan sulfate (HS), composed of repeating disaccharide units of glucosamine and hexuronic acid. HS is an important structural component of the lung parenchyma and a regulator of signaling pathways. HS degradation enables neutrophils to access endothelial adhesion molecules, extravasate, and further enhance the inflammatory lung injury [47]. Neutrophils-derived antimicrobial peptides such as cathelicidin-related antimicrobial peptide (LL-37), MPO, and histones are cytotoxic to endothelial and bronchial epithelial cells. They cause alveolar and capillary damage, resulting in ARDS development [48,49,50]. 

NE causes a slowing in the ciliary beat frequency, diminishing mucociliary transport, and resulting in disruption of bronchial epithelial cells. Moreover, NE damages the endothelial actin cytoskeleton, E-cadherin, and VE-cadherin, disrupting the mucosal barrier and increasing the permeability of the alveolar-capillary barrier [45,51,52]. Additionally, epithelial cell death mediated by NE is at least in part due to apoptosis, and it is accompanied by pro-inflammatory cytokine release via a protease-activated receptor (PAR-1) and nuclear factor kappa B (NF-κB) and p53-dependent mechanisms [53]. 

### 4.3. NETs as Triggers of Cytokine Storm in SARS-CoV-2 Disease

It is estimated that 10–15% of patients with severe COVID-19 progressed to ARDS triggered by cytokine storm, due to overproduction of IL-6, IL-8, IL-10, IL-17, and TNF-α [54]. These mediators recruit and activate a large panel of inflammatory cells, further enhancing lung tissue damage.

The analysis of clinical data revealed a markedly increased neutrophil count, lymphopenia, increased neutrophils-to-lymphocytes ratio and neutrophils-to-lymphocytes CD4+ ratio, and elevated CRP, LDH, and IL-6 concentrations in critically ill COVID-19 patients [21]. The clinical definition of cytokine storm is usually based on a finding of serum ferritin>700 ng/mL, CRP>30 mg/dL, and LDH>300 u/L [55]. 

Increased plasma D-dimer (DD) is seen in 80% of patients with signs of cytokine storm [55]. D-dimer, a product of fibrin degradation by plasmin, illustrates both the intensity of inflammatory reaction and the endogenous fibrinolysis of thrombi. Artifoni et al. found DD > 1000 ng/mL as predictive of venous thromboembolic disease (VTE) in prospectively assessed Covid-19 patients [56]. 

Furthermore, pulmonary endothelium seems to be a key target organ in SARS-CoV-2 disease, as its surface highly expresses virus-targeted receptors. Severe endothelial cell injury in the lung microvasculature is the most critical factor for COVID-19-related ARDS, significantly contributing to the increase of vascular permeability, release of pro-inflammatory mediators, accumulation and extravasation of leukocytes, activation of pro-coagulant pathways, and disruption of the alveolar-capillary barrier [57]. 

Sera from COVID-19 patients have been shown to trigger NETs formation [42]. Elevated levels of soluble markers for NETs formation, such as circulating DNA, NE activity, or MPO-DNA complexes were found in sera from COVID-19 patients and they correlate with disease severity [42,44]. Increase of serum NETs components correlates with endothelial dysfunction and microvascular thrombosis that complicate severe COVID-19, and is associated with multi-organ damage and mortality [22,58]. 

Both microbial and host components in COVID-19 patients’ sera contribute to NETs release [58]. Staats et al. postulated that specific antibodies and immune complexes formed in the serum of COVID-19 patients additionally trigger NET formation [59]. IgA2 was shown to activate immune cells and induce NETs formation. IgA2 antibodies to SARS-CoV-2 correlated with disease severity, and enabled discrimination between non-fatal and fatal outcomes in patients with severe COVID-19 disease. Furthermore, anti-SARS-CoV-2 IgA2 correlated with the presence of extracellular DNA. These data suggest that the formation of anti-SARS-CoV-2 IgA2 is a marker of more severe disease associated with NET formation, and of a poor outcome [59]. 

### 4.4. NETs and Immunothrombosis

As described above, NETs-related imbalance of coagulation and fibrinolysis resulting in thrombotic events may contribute to the severity of COVID-19 [60]. Immunothrombosis, stimulated by NETs, may develop in critically ill COVID-19 patients, despite VTE prophylaxis with heparins [21,56,61,62]. SARS-CoV-2 destroys endothelium and leads to cell apoptosis with overexpression of clotting factors VII and VIII and tissue factor, activating endogenous and exogenous coagulation pathways. At the same time, the increased expression of plasminogen activator inhibitor-1 (PAI-1) and downregulation of anti-thrombin in patients with COVID-19 can reflect the imbalance of endothelial cell function [63]. 

Activated platelets release platelet factor 4 and neutrophil-activating peptide-2, which stimulate further recruitment and activation of neutrophils and monocytes/macrophages [21]. NETs released by neutrophils compose an important milieu which contributes to the progression of the disease and promotes coagulation. A large number of endothelial cells infected with SARS-CoV-2 become activated and highly express P-selectin. Binding to P-selectin glycoprotein ligand-1 (PSGL-1) and E-selectin promotes the aggregation of neutrophils and NETs release [42]. NE can consume anticoagulant substances anti-thrombin III (ATIII) and tissue factor pathway inhibitor (TFPI), affecting the coagulation/anticoagulation balance [64].

Some authors claim that the pathological process in the lungs in the course of COVID-19 disease may progress despite viral cleavage. SARS-CoV-2 is directly responsible for the pathologic process in lungs at an early phase of COVID-19 disease; nevertheless, the dysregulation of the immune system and immunothrombosis progress in some patients, despite the elimination of the virus. A self-perpetuating vicious cycle of tissue damage is supported even in the absence of the initiating agent. SARS-CoV-2 nucleoprotein was demonstrated in type 2 pneumocytes and alveolar macrophages, as well as in hyaline membranes, in the areas of exudative DAD, but not in the fields of proliferative DAD [38]. Median duration of viral positivity in the lungs was approximately 14 days; nevertheless, viral genetic material was occasionally found in lung tissue for up to 4 weeks [37,38]. 

Different studies have highlighted the importance of immunological dysregulation caused by pathologic neutrophilic inflammation, NETs formation, and immunothrombosis. Verras et al. demonstrated the increase of NETs concentration in plasma, tracheal aspirate, and in lung tissue specimens obtained at autopsies [30]. Radermecker et al. found NETs in lung specimens of all patients who died of COVID-19 disease, and in none of the patients from the control group of non-COVID-19 lung diseases [23]. Focal occlusion of bronchioles and lung alveoli by conglomerates of NETs with fibrin was proved. Nicolai et al. demonstrated excessive platelet and neutrophil activation in SARS-CoV-2 lung autopsies in comparison with non-COVID autopsies [39]. Moreover, NETs were found in pulmonary arterioles in the areas occluded by thrombi [23,39]. In view of these observations, some authors speculate that immunothrombosis is probably the most important cause of lethality in COVID-19 disease, playing an essential role in the development of acute lung injury [24,30].

## 5. Therapeutic Approaches to NETs-Related Lung Disease in SARS-CoV-2 Infection

As NETs components strongly contribute to the development of thrombosis and inflammation, effective anti-NETs therapy could have a beneficial role in treatment of severe COVID-19 disease [60].

As an example, R406, the metabolically active component of fostamatinib, has been shown to limit pro-inflammatory cytokine production of human macrophages stimulated by COVID-19 plasma [65]. Strich et al. proved that fostamatinib significantly abrogated NETosis in healthy donor neutrophils triggered by plasma from COVID-19 patients, in a dose-dependent manner, raising the potential for therapeutic benefit of fostamatinib for severe COVID-19 [65]. 

Further attempts to influence NETs-related lung pathology focused on the use of dornase alpha, a recombinant human DNase-1. The ability of DNase to degrade NETs was confirmed in lungs of calves infected with bovine respiratory syncytial virus [66], as well as in the septic mice model [67,68]. Dornase alpha has been used for many years as a mucolytic agent in cystic fibrosis patients. The inhalation of dornase alpha (Pulmozyme) results in the dissolution of thick mucus that obliterates airways, containing the complexes of NETs, bacteria, and bronchial epithelial cells. Endogenous DNase activity is markedly reduced in the plasma of severe COVID-19 patients, compared to those with mild SARS-CoV-2 disease and healthy volunteers [67]. As the half-life of dornase alpha is very short, the new DNase formulas based on nanospheres coating are used [67,68]. The effectiveness of DNase-1-coated nanospheres was confirmed by in vivo study in a septic mice model [67,68]. The experimental use of DNase-1-coated melanin-like nanospheres on plasma of COVID-19 patients resulted in significant reduction of NETs and MPO activity, as well as the decrease of the cytokines IL-1b, IL-6, and TNFα, involved in the NETs pathological loop [67]. Nevertheless, the products of NETs degradation by DNase may stimulate bacterial growth. This was proven for *Hemophilus influenzae*, which is frequently cultured as co-infector in SARS-CoV-2 patients [66]. Thus, potential benefits of DNase-containing therapeutics in SARS-CoV-2 infection have to be confirmed by further studies. 

The other naturally occurring molecule which reduces pathological NETs activity is alpha-1-antitripsin (AAT), a serine protease inhibitor (SERPIN). AAT binds extracellular IL-8, reducing the neutrophils’ influx to the inflammatory site, and augments neutrophil superoxide production [69]. The other beneficial effects of AAT concern the inhibition of endothelial cells apoptosis and thrombin generation. These properties may be important in reducing NETosis and immunothrombosis in the course of SARS-CoV-2 infection. Moreover, AAT expresses natural anti-SARS-CoV-2 activity by the inhibition of S-protein cleavage [69]. AAT plasma concentration is increased in the course of severe infections. The individuals diagnosed with AAT deficiency were more prone to developing uncontrolled infections. Vianello and Braccioni, looking for predictors of severe SARS-CoV-2 disease in the Italian population, noticed the geographic co-localization of AAT deficiency and SARS-CoV-2 infections [70]. Thus, it is possible that the patients with severe SARS-CoV-2 disease could benefit from therapeutic AAT administration [69]. 

Another attempt to inhibit NETosis in SARS-CoV-2 disease is the use of dociparstat. Dociparstat sodium (DSAT) is a glycosaminoglycan derivative of heparin, with reduced anticoagulant activity but with maintained anti-inflammatory properties [71]. The drug is currently used in the treatment of acute myeloid leukemia. DSAT inhibits the activity of high mobility group box protein 1 (HMGB1), platelet factor 4, and P-selectin, thus influencing both NETs and platelet-leukocyte aggregates formation. Therefore, it may have potential activity against SARS-CoV-2-induced immunothrombosis. The randomized clinical trial of dociparstat in acute lung injury due to COVID-19 disease is ongoing [71]. Further clinical investigations are warranted to discern alternative approaches to the NETs-related pathology.

## 6. Final Remarks

Recently, different studies have highlighted the link between NETs and COVID-19 disease. In view of the growing number of previous studies, this is mainly attributed to pro-inflammatory and pro-thrombotic properties of NETs and its components, as well as to the direct cytotoxic effects of these structures. Nevertheless, specialists agree that the mechanisms responsible for acute lung injury and other organ dysfunction in SARS-CoV-2 infection are far from being elucidated. We highlighted the complexity of these mechanisms, with a special role of NETs in orchestrating the inflammatory processes.

Evidence has also accumulated, showing that polymorphonuclear leukocytes are able to regulate adaptive immune responses via NETs [72]. According to numerous observations, NETs participate in the direct priming of T cells and accordingly enhance inflammatory response mechanisms. However, the functional consequences of NETs interacting with specialized immune cells in SARS-CoV-2 infection require further studies.

We have underlined the role of NETs as triggers of a cascade of inflammatory reactions leading to cytokine storm during COVID-19. NETs serine proteases activate a large panel of pro-inflammatory cytokines which dramatically escalate the inflammatory reaction and change its character [73]. Further investigations will hopefully address the question of whether a better understanding of these mechanisms may inspire future diagnostic strategies and open novel therapeutic strategies for COVID-19 patients. Scientists agree that the role of NETs in the course of COVID-19 is paradoxical under certain circumstances and beneficial only if properly regulated; thus, the costs of NET formation may result in direct tissue injury and enhanced inflammation.

## Data Availability

Not applicable.
